# COVID-19 death: A novel method of improving its identification when a patient has multiple diagnoses

**DOI:** 10.4102/sajid.v37i1.349

**Published:** 2022-04-26

**Authors:** Nnabuike C. Ngene, Jagidesa Moodley

**Affiliations:** 1 Department of Obstetrics and Gynaecology, School of Clinical Medicine, Faculty of Health Sciences, University of the Witwatersrand, Johannesburg, South Africa; 2 Department of Obstetrics and Gynaecology, Leratong Hospital, Krugersdorp, South Africa; 3 Women’s Health and HIV Research Group, Department of Obstetrics and Gynaecology, Faculty of Health Sciences, University of KwaZulu-Natal, Durban, South Africa

**Keywords:** cause of death, COVID-19 death, death statistics, modified NJ model II, principal diagnosis

## Abstract

Assigning a primary cause of death to a deceased patient who had multiple principal diagnoses including coronavirus disease 2019 (COVID-19) is challenging because of the difficulty in selecting the most appropriate cause. To proffer a solution, the authors reviewed the literature on assigning a primary cause of death. In 2015, the Nnabuike-Jagidesa (NJ) model II was devised to improve the International Classification of Diseases and related health problems, 10th revision (ICD-10) guideline on how to assign a primary cause of death. The NJ model II stipulates that when there are multiple diagnoses with no plausible explanation that one of the illnesses could have resulted in the other clinical conditions, the single most appropriate primary cause of death is the condition with the highest case fatality ratio in that setting. In the index report, the authors opine that if the case fatality ratios are similar, the following objective criteria (listed in the order of priority) should be used to assign a primary cause of death: condition with the highest infection fatality ratio, condition that was the main indication for the last acute surgical or invasive procedure performed (during the course of the same ill-health) before the death and the disease that theoretically affects the highest number of body organs. Additionally, a clinical descriptor should be used when none of the objective criteria are satisfied. This novel approach, termed the *modified NJ model II*, is expected to improve the objectivity and reproducibility of the assigned primary cause of death in a deceased who had multiple diagnoses, which may include COVID-19.

## Introduction

Assigning a single primary cause of death when a deceased had multiple principal diagnoses is challenging because of the difficulty in selecting the most appropriate cause.^1^ This is of particular importance when one of the principal diagnoses is a dreadful pandemic such as the coronavirus disease 2019 (COVID-19), caused by severe acute respiratory syndrome coronavirus 2 (SARS-CoV-2) infection. Coronavirus disease 2019 is highly contagious and results in high number of human deaths because it rapidly spreads to affect many individuals. If the overall clinical condition of the deceased is not well considered, a wrong primary cause of death may be assigned.

According to the World Health Organization (WHO):

[*A*] death due to COVID-19 is defined for surveillance purposes as a death resulting from a clinically compatible illness, in a probable or confirmed COVID-19 case, unless there is a clear alternative cause of death that cannot be related to COVID disease (e.g., trauma). There should be no period of complete recovery from COVID-19 between illness and death.^2^

Notably, the plurality of possible pathways of death associated with COVID-19^3^ make it complex to determine the contribution of any co-existing clinical condition to the cause of death. Despite these complexities, the WHO recommendation on assigning death because of COVID-19 does not indicate the measures to be used to improve the reproducibility of an assigned primary cause of death. Nonetheless, the WHO method of assigning primary cause of death due to COVID-19 is thoughtful, useful and commendable but may be prone to error that has the potential to skew the mortality statistics. For instance, it does not provide for the use of objective measures such as case fatality ratio, infection fatality ratio, medical condition necessitating the acute surgical or invasive procedure before death and the disease that theoretically affects the highest number of body organs. These objective measures may improve the reproducibility of the assigned cause of death.

Of note, the infection fatality ratio or rate is the proportion of mortalities from the total number of all infected individuals. On the other hand, the case fatality ratio or rate is the proportion of deaths from the total number of confirmed cases of disease.^2^ Understandably, some death notification guidelines mention the use of opinion descriptors such as listing a cause of death as ‘probable’^4^ although with no explanation, in a stepwise use approach, about when they can be used given the availability of other objective measures. In routine clinical practice, therefore, these opinion descriptors are not usually used by many healthcare professionals in the context of COVID-19 when assigning the primary cause of death. As a result of this challenge, COVID-19 is often chosen as a cause of death in individuals who test positive for SARS-CoV-2 infection. This solitary use of laboratory confirmation of SARS-CoV-2 infection to assign COVID-19 as the primary cause of death is often practised. Understandably, the cause of death should be the best medical opinion of the medical practitioner but the method of arriving at the ‘best medical opinion’ has to be guided by the use of objective measures that can result in a reproducible cause of death if the same scenario is analysed by different clinicians. To address this challenge, a method of assigning a primary cause of death that gives due consideration to stepwise use of objective measures is required. The authors, therefore, describe the sequential use of case fatality ratio, infection fatality ratio, condition necessitating the acute event before death, the number of body organs affected by a disease and clinical descriptor or modifiers in what we term modified Nnabuike-Jagidesa (NJ) model II to assign a single most appropriate primary cause of death to a deceased who had multiple principal diagnoses, which may include COVID-19. This is a modification of a previously reported NJ model II.^1^

## Assigning a primary cause of death using the modified Nnabuike-Jagidesa model II

**FIGURE 1:**
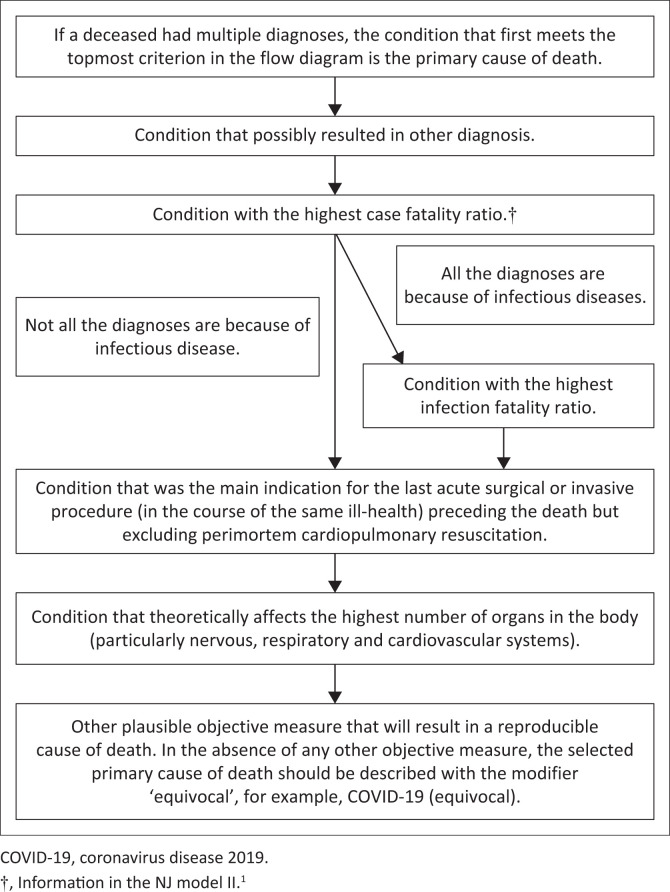
Assigning primary cause of death using the modified Nnabuike-Jagidesa model II.

In 2014, the NJ model I was developed that describes how to assign an appropriate and comprehensive diagnosis.^5^ In 2015, the NJ model II was devised, which explains how to assign an appropriate primary cause of death and indication for medical procedures.^1^ The details of how to use the NJ model II^1^ to assign a primary cause of death is shown in Figure 1. To assign the most appropriate cause of death, the NJ model II stipulates: when a primary cause of death is suspected to be any one of the multiple diagnoses, with no plausible explanation that one of the medical conditions could have resulted in the other clinical diagnosis, the single most appropriate cause of death is the condition with the highest case fatality rate, ratio or risk in that setting.^1^

Given that the case fatality rates for diseases may be similar or uncertain, there is a need to modify the NJ model II using other objective criteria. It is proposed in the index report that if the case fatality ratios are similar, the most appropriate primary cause of death is the disease with the highest infection fatality ratio in that setting. Furthermore, if the infection fatality ratios of the diseases are similar or if all the diagnoses are not because of an infection, the primary cause of death is the condition necessitating the last acute surgical or invasive procedure (during the course of the same ill-health) preceding death but excluding perimortem cardiopulmonary resuscitation, which is often a generic care. In the absence of the foregoing, the primary cause of death is the condition which theoretically affects the highest number of organs in the body. If an objective criterion is not met, a modifier such as ‘equivocal’ should be appended on the chosen cause of death, for example, COVID-19 (equivocal). The descriptive modifier appended to the primary cause of death means that the assigned cause of death is uncertain. Figure 1 also illustrates the modified NJ model II that contains the objective criteria whose order of priority in the design of the model is based substantially on pragmatism and the ability to reproduce the assigned cause of death.

## Evaluation of the modified Nnabuike-Jagidesa model II to improve death statistics

To evaluate modified NJ model II, the two methods (the modified NJ model II and the WHO recommendation on how to assign a cause of death because of COVID-19) should be used by healthcare professionals to assign the primary cause of death to deceased patients with multiple diagnoses. The frequency of occurrence of each cause of death using a particular method should be calculated. The modified NJ model II will be proven to be better than the WHO recommendation if its use results in assigning a cause of death with the highest frequency across the healthcare professionals. Currently, plans are underway to validate the NJ model II through a prospective study.

The authors recognise the need to calculate the case fatality and infection fatality ratios in different settings for effective utilistion of the modified NJ model II but this can be achieved through collection and analysis of data used for monitoring and evaluation. Furthermore, laboratory confirmation of SARS-CoV-2 infection is essential for the diagnosis of COVID-19.^6^ Given that the commonly used SARS-CoV-2 real-time reverse transcription polymerase chain reaction (RT-PCR) test is over 95% specific^7^ with a false negative rate of 2% – 30%,^8,9^ strong clinical suspicion of COVID-19 death based on clinically compatible illness may be a replacement for ‘laboratory confirmation of SARS-CoV-2 infection’ when using the modified NJ model II.

There are obstacles that are encountered in calculating measures such as case fatality ratio, infection fatality ratio, etc. in low- and middle-income countries such as South Africa. These include difficulties in establishing the numerator and denominator for the calculation of the fatality and mortality rates. For instance, as it concerns SARS-CoV-2, many individuals with the infection show little or no symptoms. Amongst those who are symptomatic, there are limited access to laboratory testing and medical intervention to improve outcomes particularly in the rural areas. Another challenge is that the fatality ratio may be influenced by other variables such as age and gender and this requires disaggregated or sub-group calculations.^10^ Additionally, the issue of excess mortality attributable to COVID-19 is challenging in settings where data collection is inadeqaute.

## Clinical vignette on how to assign a primary cause of death

Given the concerns about the large number of deaths caused by COVID-19 in different settings worldwide,^11,12^ emphasis has been placed on the need to correctly assign and report COVID-19 deaths.^13,14^ Similar to practices elsewhere in the world, the cause of death assigned to a deceased in South Africa is based on the best opinion of the attending clinician. In clinical practice, when a death occurs, the attending physician issues a death certificate, which contains the underlying cause of death. In addition, the daily statistics on causes of deaths in many healthcare facilities is based on the same underlying causes of deaths assigned by the attending healthcare professionals. Usually, no further revision is made to the assigned cause of death written in the death certificate if a mistake is discovered later. This practice resonates with the reports of other experts on the mistruths about the number of COVID-19 deaths.^12^ In the United Kingdom for instance, overestimation of the number of COVID-19 deaths has been reported and was attributed to be caused by the challenges associated with counting COVID-19 death using the deaths occurring within 28 days of a positive COVID-19 test and using the underlying cause of death written in death certificates.^15^ Ngcobo et al., in South Africa have reported that the inability to differentiate those who die with SARS-CoV-2 from those who die from SARS-CoV-2 affects the number of COVID-19 deaths.^14^

Notably, it is common to assign COVID-19 as the primary cause of death in the clinical scenario (discussed in the next paragraph) complicated by obstetric haemorrhage in sub-Saharan Africa. Notably, massive obstetric haemorrhage is a condition that has a case fatality ratio of 2.8% – 27.3% in sub-Saharan Africa,^16^ and its contemporaneous occurrence with COVID-19, which has a case fatality rate of 3.4% in sub-Saharan Africa^10^ in a deceased patient make it difficult to discern the main primary cause of death.

A pregnant woman who developed antepartum haemorrhage from placenta previa was diagnosed with COVID-19 pneumonia on admission to the hospital. Subsequently, the patient had a massive postpartum haemorrhage during an emergency caesarean delivery for the antepartum haemorrhage, received transfusion of red blood cell concentrate and was admitted to the intensive care unit because of respiratory and cardiovascular failures but died 48 h post-delivery as a result of cardiopulmonary arrest. Using the modified NJ model II, the cause of death in this case is placenta previa because the bleeding from the placenta previa was the main indication for the last acute surgical procedure (the caesarean delivery) preceding the death. This clinical scenario is important in a country such as South Africa where obstetric haemorrhage is the third commonest cause of maternal deaths, accounting for 15.7% of cases^17^ and being third only to non-pregnancy related infection (particularly HIV) and hypertensive disorders of pregnancy.

## Implication for practice

Improvement in objectivity and reproducibility associated with assigning the cause of death may be achieved by the use of the modified NJ model II. The authors envisage that the model will also improve the statistics on causes of deaths assigned to COVID-19 or any other highly contagious epidemic. Therefore, the model may assist in resolving the medical and political debate about the number of deaths caused by COVID-19 in different settings across the world. Of note, the highly contagious nature of SARS-CoV-2^18^ often deters many healthcare facilities, for example, in South Africa from performing autopsy on a deceased patient diagnosed with COVID-19. Therefore, to establish the underlying cause of death in forensic epidemiology,^19^ particularly in complex cases, the modified NJ model II may assist with improving the method of analysing the cause of death.
